# Shaping the Future of Microbial Therapies Through Intelligent Probiotic and Postbiotic Delivery

**DOI:** 10.3390/biom15121681

**Published:** 2025-12-02

**Authors:** Virginia Fuochi

**Affiliations:** Department of Biomedical and Biotechnological Sciences (BIOMETEC), University of Catania, 95123 Catania, Italy; virginia.fuochi@unict.it

The rapid expansion of microbiota research continues to influence how we interpret human physiology and the mechanisms underlying disease. Once regarded primarily as modulators of gastrointestinal balance, prebiotics, probiotics, and related microbial products are now recognized as elements capable of shaping systemic processes ranging from immune homeostasis to neurobehavioral regulation. The contributions gathered in this Special Issue, “Prebiotics and Probiotics in Health and Disease: Looking at the Future”, reflect this conceptual transition, illustrating how microbiota-centered approaches intersect with immunology, metabolism, neuroscience, dermatology, oncology, and translational animal research [1,2,3,4,5,6,7,8].

A unifying theme emerging across these works is the dynamic role of the gut ecosystem in regulating host responses under both physiological and pathological conditions. Several articles examine how microbial signaling influences the balance between inflammatory and regulatory pathways, highlighting microbiota-mediated modulation of cytokine networks, antigen presentation, and epithelial resilience [1,2,7,8]. These observations align with the broader scientific understanding that microbial metabolites, surface molecules, and membrane-associated structures contribute substantially to shaping local and systemic immunity.

The interface between microbial signals and host neurobiology also receives growing attention, with evidence supporting a bidirectional communication along the microbiota–gut–brain axis. Behavioral outputs, stress-related responses, and neuroinflammation can be influenced by perturbations in microbial composition or function, as illustrated by avian models linking cecal microbiota to stress-induced injurious behavior [3]. Neurodegenerative conditions such as Parkinson’s disease are likewise increasingly studied through the lens of intestinal dysbiosis, altered intestinal permeability, and microglial activation, indicating that gastrointestinal ecosystems may participate in early or exacerbating phases of neurological disorders [4].

Inflammatory and autoimmune diseases provide another area where microbiota–host interactions appear particularly relevant. Dysbiosis, altered metabolite production, and impaired mucosal barrier function emerge as co-factors in the onset and progression of chronic inflammatory conditions, including rheumatoid arthritis [2]. The mechanistic diversity of these interactions underscores why certain probiotic strains, but not others, exert beneficial effects, and why structural features, such as bile salt hydrolase activity or surface-associated immunomodulatory molecules, may determine strain-specific functions and clinical responses [1,5,7].

Beyond human disease, it is important to emphasize the relevance of translational models. Porcine systems provide valuable insights into dietary modulation, gastrointestinal physiology, and immune responses, given their close resemblance to human biology [8]. Such models help bridge mechanistic studies with future clinical applications and allow controlled evaluation of prebiotic and probiotic interventions. Avian models, in turn, offer alternative routes to explore the relationship between microbial communities and behavioral or neuroendocrine outputs, with implications that extend beyond animal welfare [3].

Cutaneous and mucosal environments are similarly influenced by gut-derived microbial signals, as seen in conditions where immune regulation and neurovascular dynamics intersect. Diet, microbial metabolites, and targeted supplementation strategies appear capable of modulating inflammatory responses well beyond the intestinal tract, suggesting that the gut–skin axis is an expanding field with therapeutic implications in disorders such as rosacea [6]. In parallel, in vitro and in vivo models show how specific probiotic formulations can interfere with tumor cell signaling, limiting proliferation and migration, thereby providing a rationale for exploring probiotic adjuvants in cancer therapy [5].

Complementing these broader perspectives, these contributions presented concrete scientific advancements that strengthen the emerging framework of microbial therapies. The original research by Agolino et al. identifies two *Lacticaseibacillus rhamnosus* strains with distinct bile salt hydrolase activity and stress tolerance, supporting the rational selection of probiotic candidates based on enzymatic and genomic signatures [1]. The work highlights how strain-level genomic differences translate into functional variations relevant for host metabolic regulation.

Moreover, in the context of autoimmune disorders, Chasov et al. provide an updated systematic framework linking intestinal dysbiosis to rheumatoid arthritis, emphasizing microbial metabolites and mucosal immunity as potential therapeutic entry points [2].

Furthermore, the study by Fu and Cheng demonstrates that cecal microbiota transplantation reshapes stress-driven behavioral phenotypes in poultry, offering a unique translational model to explore microbiota-mediated neurobehavioral modulation [3].

Neurogastroenterology is further advanced by Gabrielli et al., who consolidate evidence for microbial contributions to α-synuclein pathology, intestinal permeability, and microglial activation in Parkinson’s disease, presenting an updated synthesis that connects gastrointestinal alterations with neurodegenerative processes [4]. In oncology, Giorgi et al. demonstrate that selected probiotic formulations modulate cell-cycle regulators, migration pathways, and oncogenic signaling in female cancer cell lines, providing a rationale for microbiome-based adjuvant therapies [5]. The review by Manfredini et al. links gut dysbiosis, diet, and neurovascular inflammation to rosacea pathogenesis, outlining how microbial and nutritional interventions may reshape cutaneous immune homeostasis [6]. Complementing these human-centered perspectives, Rossi and Mainardi highlight the translational relevance of porcine models, detailing how controlled prebiotic and probiotic interventions in pigs can mirror human immune, metabolic, and microbial responses [8]. Finally, Rossi et al. provide an in-depth analysis of dairy propionibacteria, elucidating how their surface proteins, metabolites, and extracellular vesicles contribute to immunomodulation, antimicrobial activity, and metabolic benefits [7].

Together, these studies contribute specific biologically grounded understanding, identify strain-resolved functional traits, and introduce translational models that enhance the precision and predictability of microbiota-based interventions.

Microbial-derived molecules themselves are central to this emerging landscape. As highlighted across several contributions in this Special Issue, many of the observed immunological and metabolic effects can be traced back to specific microbial effectors rather than to the presence of whole cells. Surface layer proteins, extracellular vesicles, and metabolites such as short-chain fatty acids contribute to immunomodulatory, metabolic, and barrier-supporting outcomes, helping explain the strain- and species-specific functions described in recent studies. Additionally, antimicrobial peptides and bacteriocins, whose relevance in food safety, microbial ecology, and host protection has been extensively reviewed elsewhere [9], represent an expanding class of bioactive products with potential applications that extend beyond traditional probiotic concepts. Looking ahead, a central challenge is not only what microbial products to employ, but how, where, and when to deliver them. Many of the limitations that currently constrain the translation of probiotic and postbiotic interventions, such as instability in the upper gastrointestinal tract, poor mucosal retention, off-target effects, or the need for high systemic doses, mirror longstanding problems in the drug delivery field. In recent decades, lipid-based and nanosized carriers have been widely explored to improve the pharmacokinetic and tissue-targeting profiles of anti-infective agents, including antibiotics and antifungals [10,11]. The same engineering principles can now be repurposed to design “smart” vehicles for biotics, shifting from simple oral supplementation toward controlled, site-specific modulation of the microbiota–host interface.

In this context, encapsulation and nanoencapsulation technologies for probiotic delivery have gained considerable momentum. Recent reviews describe polymeric, lipid-based, and hybrid nanocarriers capable of protecting viable probiotics during processing and gastrointestinal transit, while enabling controlled release in the intestine [12,13,14]. Additional evidence highlights how micro- and nanoencapsulation platforms improve gastrointestinal survival and functional delivery across diverse probiotic taxa [15]. Moreover, single-cell “armor” systems and advanced encapsulation strategies further enhance resistance to acid, bile, and mechanical stress, and allow more precise colon-targeted delivery [12,14]. Furthermore, recent technological assessments also confirm the feasibility of micro- and nanoscale coatings for protecting probiotic cells under harsh physicochemical conditions [16]. Beyond improving viability, these platforms open the possibility of co-delivering probiotics with prebiotics, micronutrients, or conventional drugs, creating integrated combination products in which microbial and pharmacological components act in a coordinated manner [13,17].

For postbiotics, the opportunities for drug delivery may be even broader. Because postbiotics consist of defined metabolites, macromolecules, or vesicles rather than live cells, they are inherently compatible with a wide range of formulation strategies. Comprehensive reviews have outlined the main in vivo routes and formulation constraints for postbiotic administration, emphasizing the need to tailor delivery to target tissues and desired pharmacodynamic profiles [18,19,20,21]. Nanoparticle-based carriers can enhance the stability and bioavailability of postbiotic molecules, improve their accumulation at inflamed or neoplastic sites, and reduce systemic exposure. Recent preclinical studies have used colon-targeted engineered postbiotic nanoparticles and other nanostructured systems to concentrate bioactive molecules at the diseased mucosa, achieving enhanced anti-inflammatory and antitumor efficacy in animal models [20,22].

This convergence between microbiome science and advanced drug delivery systems is now moving toward precision strategies. On one side, detailed characterization of probiotic and postbiotic mechanisms is expanding the catalog of bioactive signals that could be therapeutically exploited [4,5,7,19,21]. On the other, the drug delivery field is increasingly focused on responsive, tissue-targeted, and patient-tailored carriers, including AI-assisted design of nanosystems and formulation optimization. Conceptual frameworks have been proposed in which well-defined microbial molecules, such as indole derivatives and other postbiotic mediators, are coupled with AI-guided delivery platforms to achieve spatiotemporally controlled interventions [23]. Together with emerging concepts such as probiotic-based nanoparticles and engineered microbial chassis [17], these trends suggest that future probiotics, synbiotics, and postbiotics will be formulated not simply as dietary supplements but as rationally engineered, microbiota-informed therapeutics.

Collectively, the expanding body of microbiome research demonstrates a clear shift from empirical observations toward biologically grounded and technologically integrated approaches. The integration of molecular microbiology, host immunology, neuroscience, oncology, and translational modeling is progressively enabling the rational design of targeted microbial interventions. Probiotics and prebiotics are now evaluated not only as modulators of gut composition but as tools capable of influencing disease susceptibility, inflammatory tone, barrier function, and neuroimmune regulation. As the field advances, better standardization, rigorous clinical validation, and strain-level as well as molecule-level functional characterization will be essential to transform these insights into reproducible and safe therapeutic strategies.

## Data Availability

No new data were created or analyzed in this study. Data sharing is not applicable to this article.
